# Supplementary material for: VespAI: a deep learning-based system for the detection of invasive hornets
Source: Commun Biol. 2024 Apr 3;7:354. doi: 10.1038/s42003-024-05979-z (PMC10991484; doi:10.1038/s42003-024-05979-z)
Supplement: Supplementary file 2 — Supplementary Information [file 42003_2024_5979_MOESM2_ESM.pdf]

VespAI: A Deep Learning-Based System for the Detection of Invasive Hornets

Thomas A. O'Shea-Wheller, Andrew Corbett, Juliet L. Osborne, Mario Recker, and Peter J. Kennedy

Supplementary Information

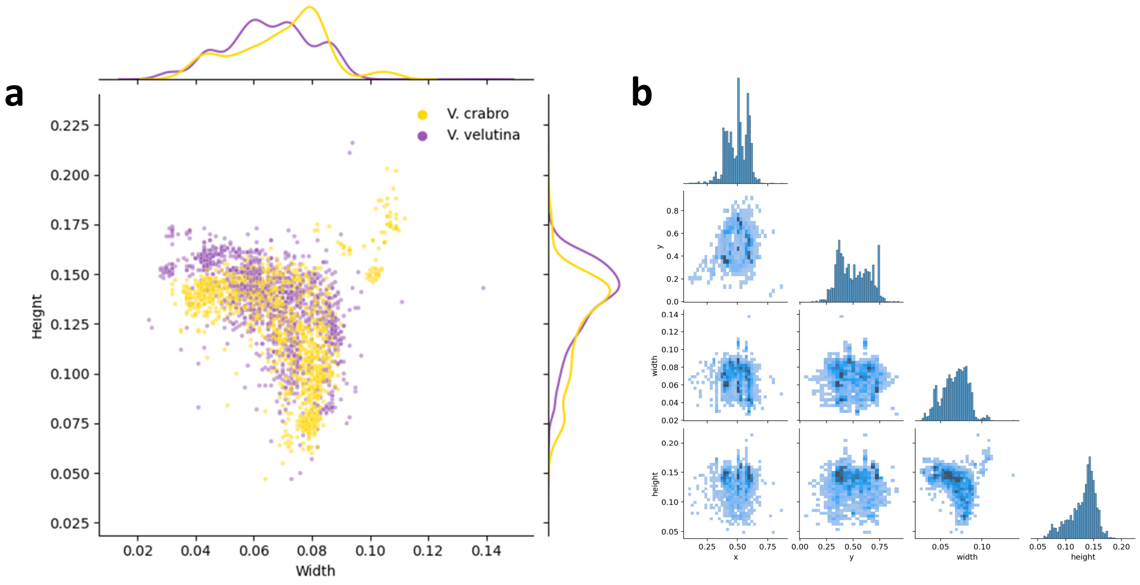

Fig. S1. (a) Dimensions of bounding boxes from hornet detections, coloured by species (*V. crabro*, yellow; *V. velutina*, purple) ($N_{V.\ crabro}$=1481, $N_{V.\ velutina}$=1545). Values indicate width and height as a proportion of the total frame size. Marginal axes display kernel density estimates for each dimension, divided by species. This data was used to refine pre-filter parameters in ViBe, ensuring that only objects within the possible hornet size range would be passed on to the YOLOv5 object detection and classification algorithm. (b) Dimensions of bounding boxes from all hornet detections correlated against hornet locations in frame, coloured by density (low, light blue; high, dark blue) (*N*=3026). Values indicate width and height as a proportion of the total frame size, and x and y coordinates within images. Marginal axes display density estimates for each dimension and coordinate. This data was used to confirm hornet size ranges across species, and evaluate detection locations within images.

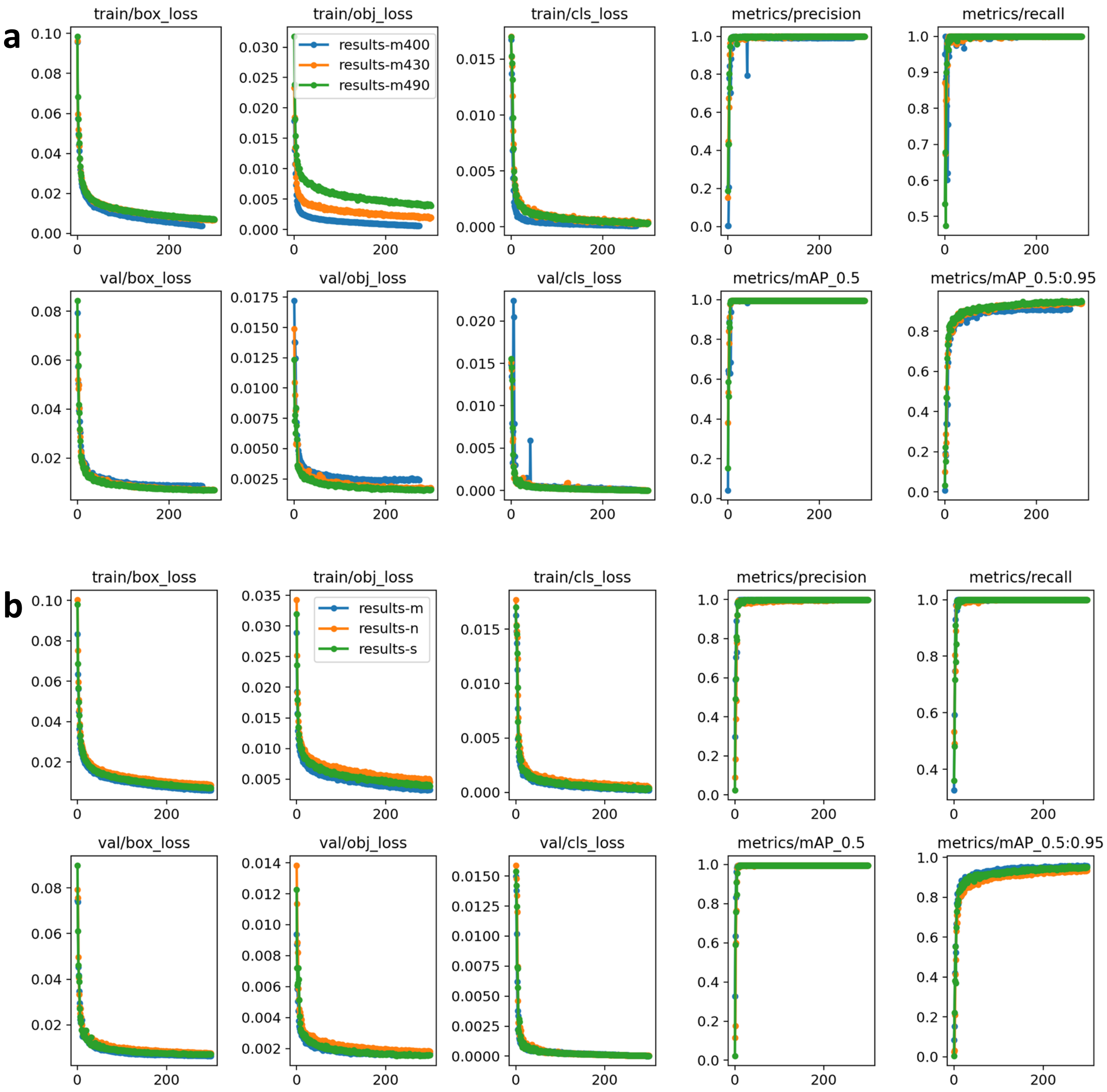

Fig. S2. (a) Model performance comparisons for increasing degrees of data augmentation. Line colours indicate degree of augmentation (0%, blue; 30%, orange; 90%, green) (*N*=15). (b) Model performance comparisons for YOLOv5m, YOLOv5s, and YOLOv5n architectures (*N*=15). Line colours denote model architecture (YOLOv5m, blue; YOLOv5s, green; YOLOv5n, orange) ranked by model size from largest to smallest. Panels from left to right display box loss, indicating how effectively a model locates the centre of a hornet and thus constructs a bounding box; objectness loss, indicating how likely the proposed region of interest is to contain a hornet; classification loss, indicating how well the model predicts the class of a hornet (*V. velutina* or *V. crabro*); precision, indicating the proportion of hornet detections that are correct; and recall, indicating the proportion of all possible hornets that are detected.

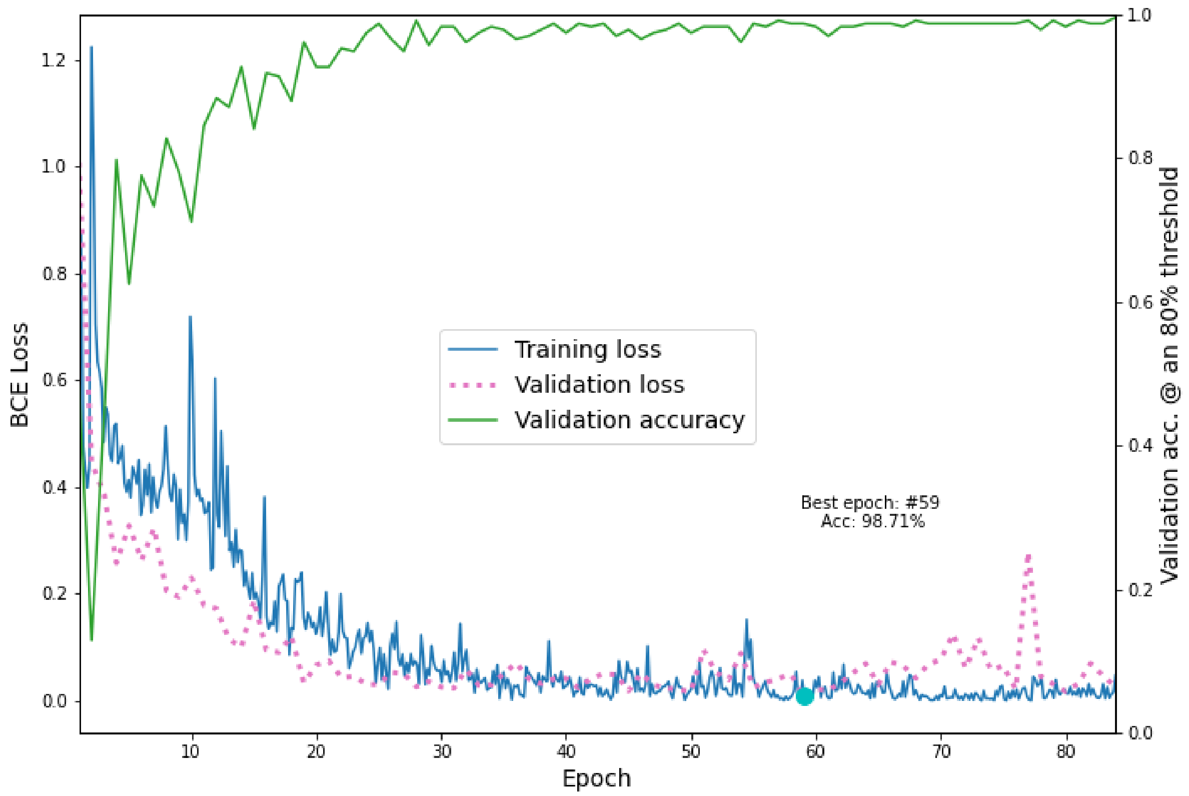

Fig. S3. Training and validation performance for the independent LRP classification model. This model employed a ResNet18 classifier trained on the same data as the YOLOv5s classification model, and served to independently validate the system via LRP outputs. Results were compared to those obtained from the end-to-end YOLOv5s LRP outputs, thus confirming equivalence. Line colours and types indicate model performance metrics (training loss, solid blue; validation loss, dashed pink; validation accuracy, solid green), and the blue circle denotes the training epoch at which optimal performance was achieved.

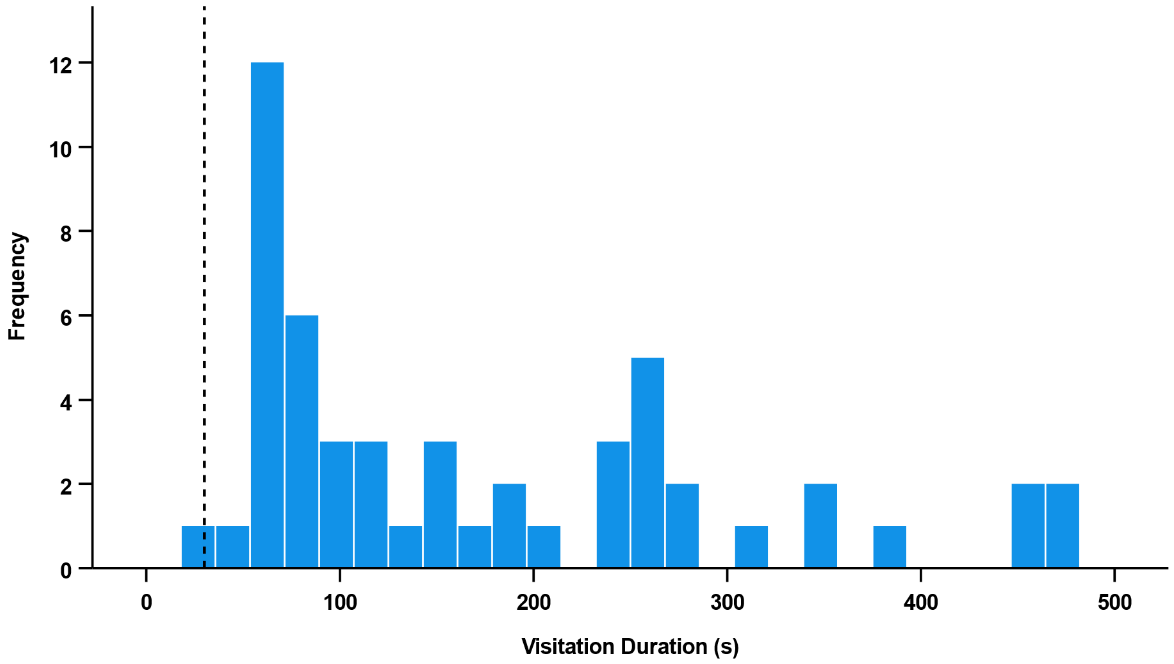

Fig. S4. *V. velutina* visit duration frequency distribution for sampled bait stations (*N*=52). The dashed vertical line indicates the shortest recorded visit duration, this being the minimum time that a hornet spent feeding at a bait station. This was subsequently used to determine the maximum prospective frame collection interval for use during field testing of the prototype monitor.

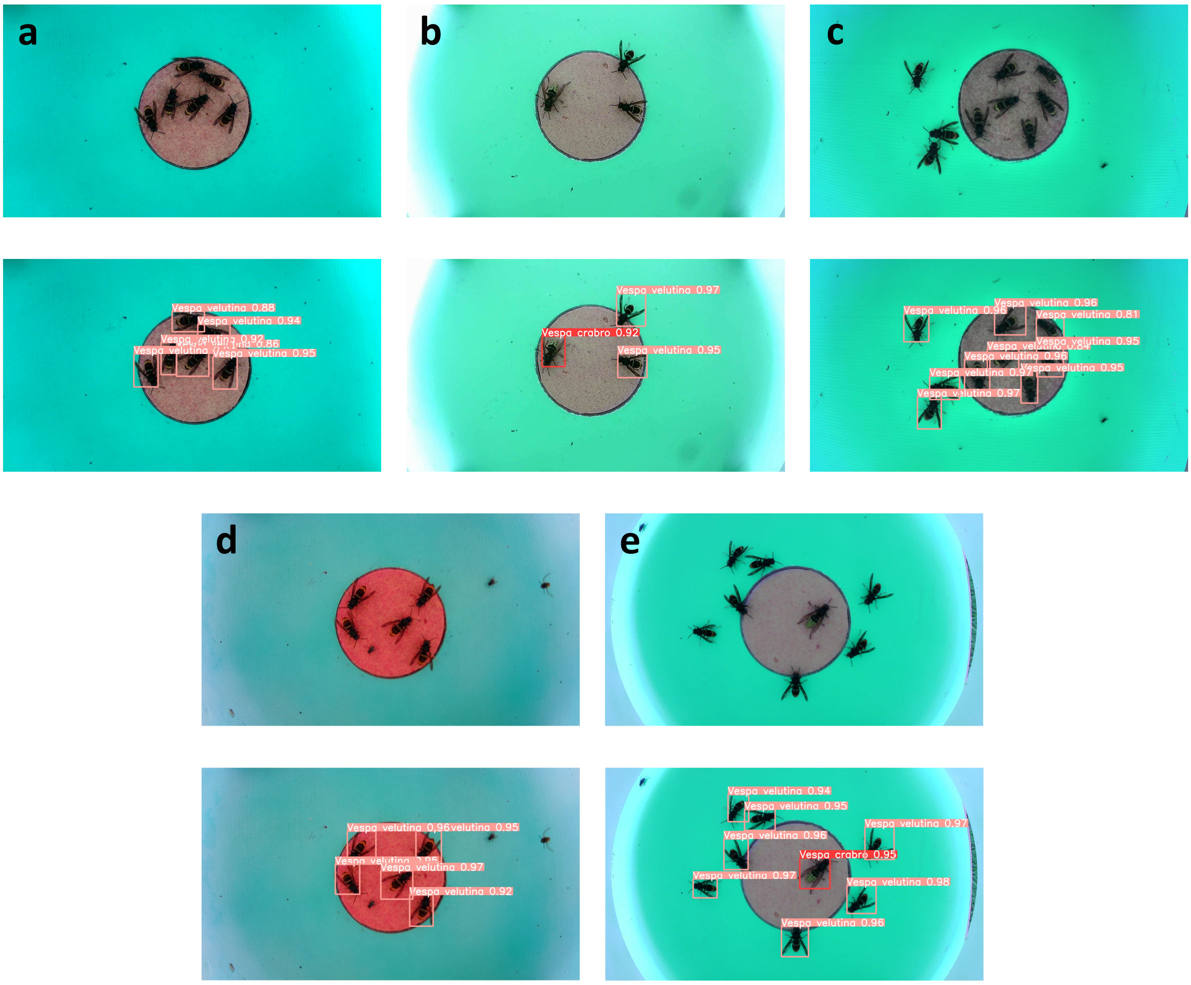

Fig. S5. Example images and detections from each of the camera models tested with the prototype system. These consisted of the (a) CANYON CNS-CWC5; (b) Dragon Touch Vision 1; (c) IRARUCW USB Webcam; (d) NexiGo N60; and (e) Arducam B0205. All images were collected during field trials in Jersey at a resolution of 1920x1080, with cameras being calibrated as detailed in (Table S2).

Table S1. Summary of non-target insect taxa observed visiting bait stations and incorporated into the training data.

Order	Genus	Frequency	Location
Hymenoptera	*Apis*	Low	Jersey, Portugal
Hymenoptera	*Bombus*	Low	UK, Jersey
Diptera	*Calliphora*	Low	UK
Hymenoptera	*Dolichovespula*	Intermediate	UK
Hymenoptera	*Formica*	Intermediate	Jersey
Hymenoptera	*Lasius*	Intermediate	UK, Jersey
Diptera	*Lucilia*	Intermediate	UK
Coleoptera	*Paracorymbia*	Low	Jersey
Diptera	*Pollenia*	Intermediate	UK, Jersey, France
Hymenoptera	*Polistes*	Low	Portugal, Jersey
Lepidoptera	*Vanessa*	Low	UK
Hymenoptera	*Vespula*	High	UK, Jersey, Portugal

Frequency indicates how commonly taxa were present in training data (low, 1-20 observations; intermediate, >20-100 observations; high, >100 observations).

Table S2. Summary of camera models tested with the prototype system during field trials.

Camera	Resolution	Data Format	Lens Field of View (°)	Sensor Type	Calibration Height (mm)
**CANYON CNS-CWC5**	1920x1080	MJPG/YUV	65	CMOS	245
**Dragon Touch Vision 1**	1920x1080	MP4	170	CMOS	210
**IRARUCW USB Webcam**	1920x1080	MJPG	120	BSI CMOS	235
**NexiGo N60**	1920x1080	YUY2/MJPG	110	CMOS	240
**Arducam B0205**	1920x1080	YUY2/MJPG	180	OV2710	180

Calibration height values indicate the optimal working distances of cameras when mounted at bait stations.

Table S3. Summary of field test types and results for each species during trials of the prototype system.

Test Type	Description	Trials	*Vespa velutina*			*Vespa crabro*		
			**Total**	**Precision**	**Recall**	**Total**	**Precision**	**Recall**
**All Frames Test**	100 frames utilising an artificial hornet to ensure that all frames are captured while recording only live hornet and insect visits	20	9239	0.999	0.948	350	1	0.886
**5s Test**	100 frames with a minimum frame interval of 5s	11	8420	0.999	0.909	222	1	0.868
**5s Test Alternate**	100 frames with a minimum frame interval of 5s and an alternate baffle design	3	671	0.991	0.986	71	0.992	0.983
**30s Test**	100 frames with a minimum frame interval of 30s	15	5052	0.998	0.952	396	0.996	0.879
**30s Test 24h**	100 frames with a minimum frame interval of 30s, sampled from a continuous running period of >24h	2	698	1	0.920	59	0.981	0.881
**5s Test ViBe**	100 frames with a minimum frame interval of 5s and ViBe enabled	4	2296	1	0.904	0	-	-

Trial values indicate the total number of replicates conducted for each test type, total values indicate the total number detectable hornet instances for each species.
