# Supplementary material for: VespAI: a deep learning-based system for the detection of invasive hornets
Source: Commun Biol. 2024 Apr 3;7:354. doi: 10.1038/s42003-024-05979-z (PMC10991484; doi:10.1038/s42003-024-05979-z)
Supplement: Supplementary file 3 — Description of Additional Supplementary Files [file 42003_2024_5979_MOESM3_ESM.pdf]

Description of Additional Supplementary Files

File name: Supplementary Data

Description: Source data underlying (Fig. 4a-c), collected during field trials of the VespAI system in Jersey.
