# Supplementary material for: VespAI: a deep learning-based system for the detection of invasive hornets
Source: Commun Biol. 2024 Apr 3;7:354. doi: 10.1038/s42003-024-05979-z (PMC10991484; doi:10.1038/s42003-024-05979-z)
Supplement: Supplementary file 5 — Reporting Summary [file 42003_2024_5979_MOESM5_ESM.pdf]

nature portfolio

Corresponding author(s): Thomas O'Shea-Wheller

Last updated by author(s): Feb 2, 2024

Reporting Summary

Nature Portfolio wishes to improve the reproducibility of the work that we publish. This form provides structure for consistency and transparency in reporting. For further information on Nature Portfolio policies, see our Editorial Policies and the Editorial Policy Checklist.

Statistics

For all statistical analyses, confirm that the following items are present in the figure legend, table legend, main text, or Methods section.

n/a	Confirmed	
☐	☒	The exact sample size (*n*) for each experimental group/condition, given as a discrete number and unit of measurement
☒	☐	A statement on whether measurements were taken from distinct samples or whether the same sample was measured repeatedly
☐	☒	The statistical test(s) used AND whether they are one- or two-sided<br>*Only common tests should be described solely by name; describe more complex techniques in the Methods section.*
☒	☐	A description of all covariates tested
☒	☐	A description of any assumptions or corrections, such as tests of normality and adjustment for multiple comparisons
☒	☐	A full description of the statistical parameters including central tendency (e.g. means) or other basic estimates (e.g. regression coefficient) AND variation (e.g. standard deviation) or associated estimates of uncertainty (e.g. confidence intervals)
☒	☐	For null hypothesis testing, the test statistic (e.g. *F*, *t*, *r*) with confidence intervals, effect sizes, degrees of freedom and *P* value noted<br>*Give P values as exact values whenever suitable.*
☒	☐	For Bayesian analysis, information on the choice of priors and Markov chain Monte Carlo settings
☒	☐	For hierarchical and complex designs, identification of the appropriate level for tests and full reporting of outcomes
☒	☐	Estimates of effect sizes (e.g. Cohen's *d*, Pearson's *r*), indicating how they were calculated

Our web collection on statistics for biologists contains articles on many of the points above.

Software and code

Policy information about availability of computer code

Data collection	VespAI code and Python (release v. 3.9.12).
Data analysis	SPSS (release v. 28.0.1.1) and Python (release v. 3.9.12).

For manuscripts utilizing custom algorithms or software that are central to the research but not yet described in published literature, software must be made available to editors and reviewers. We strongly encourage code deposition in a community repository (e.g. GitHub). See the Nature Portfolio guidelines for submitting code & software for further information.

Data

Policy information about availability of data

All manuscripts must include a data availability statement. This statement should provide the following information, where applicable:

- Accession codes, unique identifiers, or web links for publicly available datasets
- A description of any restrictions on data availability
- For clinical datasets or third party data, please ensure that the statement adheres to our policy

All model code, supporting data, manuals, and hardware setup instructions are available under a CC BY-NC 4.0-SA license at: https://github.com/andrw3000/vespai. This permits usage and adaptation for non-commercial applications, with any derivatives falling under the same restrictions. Access to this data must be requested via contacting the corresponding author, and providing a statement outlining its intended use case. This pathway aims to prevent unauthorised commercial usage, while facilitating research collaboration. All such requests will receive a response within 14 days.

Human research participants

Policy information about studies involving human research participants and Sex and Gender in Research.

Reporting on sex and gender	*Use the terms sex (biological attribute) and gender (shaped by social and cultural circumstances) carefully in order to avoid confusing both terms. Indicate if findings apply to only one sex or gender; describe whether sex and gender were considered in study design whether sex and/or gender was determined based on self-reporting or assigned and methods used. Provide in the source data disaggregated sex and gender data where this information has been collected, and consent has been obtained for sharing of individual-level data; provide overall numbers in this Reporting Summary. Please state if this information has not been collected. Report sex- and gender-based analyses where performed, justify reasons for lack of sex- and gender-based analysis.*
Population characteristics	*Describe the covariate-relevant population characteristics of the human research participants (e.g. age, genotypic information, past and current diagnosis and treatment categories). If you filled out the behavioural & social sciences study design questions and have nothing to add here, write "See above."*
Recruitment	*Describe how participants were recruited. Outline any potential self-selection bias or other biases that may be present and how these are likely to impact results.*
Ethics oversight	*Identify the organization(s) that approved the study protocol.*

Note that full information on the approval of the study protocol must also be provided in the manuscript.

Field-specific reporting

Please select the one below that is the best fit for your research. If you are not sure, read the appropriate sections before making your selection.

☐ Life sciences ☐ Behavioural & social sciences ☒ Ecological, evolutionary & environmental sciences

For a reference copy of the document with all sections, see nature.com/documents/nr-reporting-summary-flat.pdf

Ecological, evolutionary & environmental sciences study design

All studies must disclose on these points even when the disclosure is negative.

Study description	The study aimed to assess the efficacy of the VespAI system in detecting Vespa velutina and Vespa crabro in the field. To achieve this, four prototype systems were distributed across two sites, and assessed using a total of 55 experimental trials.
Research sample	Foraging Vespa velutina and Vespa crabro workers at two sites in Jersey, channel islands.
Sampling strategy	The prototype systems were tested for a total of 55 trials, each consisting of a ≥100-frame test in which foraging hornets were allowed to visit, and resultant images recorded.
Data collection	T.A.O.-W and P.J.K collected the data. Images of hornet detections were recorded automatically by the VespAI systems, and then manually validated by T.A.O.-W and P.J.K.
Timing and spatial scale	Experimental sampling was initiated on the 23rd of August 2023, and concluded on the 31st of August 2023. Sampling occurred daily across equally spaced intervals, with the exception of periods in which whether conditions were inclement. All sampling occurred at two sites, situated ~5km apart on the island of Jersey, channel islands.
Data exclusions	Data from cameras or systems that were misaligned was excluded from analyses. The decision to exclude such data was made a priori, as it was not feasible for use in further model training.
Reproducibility	Experimental trials were repeated across both field sites, and at a further site in Cornwall, UK, to ensure concordance between results.
Randomization	As the organisms in our study were foraging hornets from wild colonies, we had no control over the grouping or assortment of either species. Instead, we alternated between field sites during trials, as the ~5km distance between these ensured that different hornets were present at each. Beyond this, further randomisation was not necessary, as the VespAI system is intended for use in scenarios in which hornet identity and abundance is highly variable, thus our experimental trials aimed principally to replicate this.
Blinding	Blinding during data collection at field sites was achieved by automation of the sampling process, ensuring that the VespAI systems alone made decisions regarding which data was collect.

Did the study involve field work? ☒ Yes ☐ No

Field work, collection and transport

Field conditions	Fieldwork occurred in Jersey, channel islands, from the 23rd of August 2023, to the the 31st of August 2023. Field sites were located within a patchwork of natural and agricultural land cover, specifically within foraging range of Vespa velutina colonies. Average daily temperatures were ~18°C, and average monthly rainfall was ~111mm.
Location	Jersey, channel islands, 49.189793, -2.070384 (DD).
Access & import/export	Importation of relevant equipment was in compliance with UK regulations, as imports to Jersey are free from customs duty due to its status as a self-governing British Crown Dependency. Prior to study initiation, permission to conduct the work was obtained from the Government of Jersey, and from all private and commercial land owners on whose property field sites resided.
Disturbance	The establishment of bait stations to attract hornets posed a potential disturbance hazard to humans. To mitigate this, we informed land owners of their locations, and ensured that all such bait stations were placed in secluded areas at a safe distance from human activity.

Reporting for specific materials, systems and methods

We require information from authors about some types of materials, experimental systems and methods used in many studies. Here, indicate whether each material, system or method listed is relevant to your study. If you are not sure if a list item applies to your research, read the appropriate section before selecting a response.

Materials & experimental systems

n/a	Involved in the study	
☒	☐	Antibodies
☒	☐	Eukaryotic cell lines
☒	☐	Palaeontology and archaeology
☐	☒	Animals and other organisms
☒	☐	Clinical data
☒	☐	Dual use research of concern

Methods

n/a	Involved in the study	
☒	☐	ChIP-seq
☒	☐	Flow cytometry
☒	☐	MRI-based neuroimaging

Animals and other research organisms

Policy information about studies involving animals; ARRIVE guidelines recommended for reporting animal research, and Sex and Gender in Research

Laboratory animals	The study did not involve laboratory animals.
Wild animals	Vespa velutina and Vespa crabro were observed in the field, with all such observations occurring via automated image capture during visits to bait stations. As this process was passive, and conducted via the VespAI systems, no disturbance of the hornets occurred in the wild.
Reporting on sex	As hornets are eusocial hymenopterans, and thus express haplodiploidy, all workers observed during the study were female. This was considered acceptable during VespAI prototype testing, as females are likely to be the predominant visitors to bait stations during the initial stages of an invasion, with males emerging later in the season from established colonies.
Field-collected samples	The study did not involve samples collected from the field.
Ethics oversight	Guidance was provided by the University of Exeter Research Ethics and Governance team. Specifically, the study received ethics approval from the University of Exeter, and was assigned the application reference: eCORN000212 v2.0.

Note that full information on the approval of the study protocol must also be provided in the manuscript.
